# Supplementary material for: Association between oral microbiome and seven types of cancers in East Asian population: a two-sample Mendelian randomization analysis
Source: Front Mol Biosci. 2023 Nov 21;10:1327893. doi: 10.3389/fmolb.2023.1327893 (PMC10702768; doi:10.3389/fmolb.2023.1327893)
Supplement: Supplementary file 1 [file DataSheet1.ZIP › Supplementary Materials/MR plots of saliva/saliva═╝/Breast cancer/pheno.256_to_breast cancer_forest.pdf]

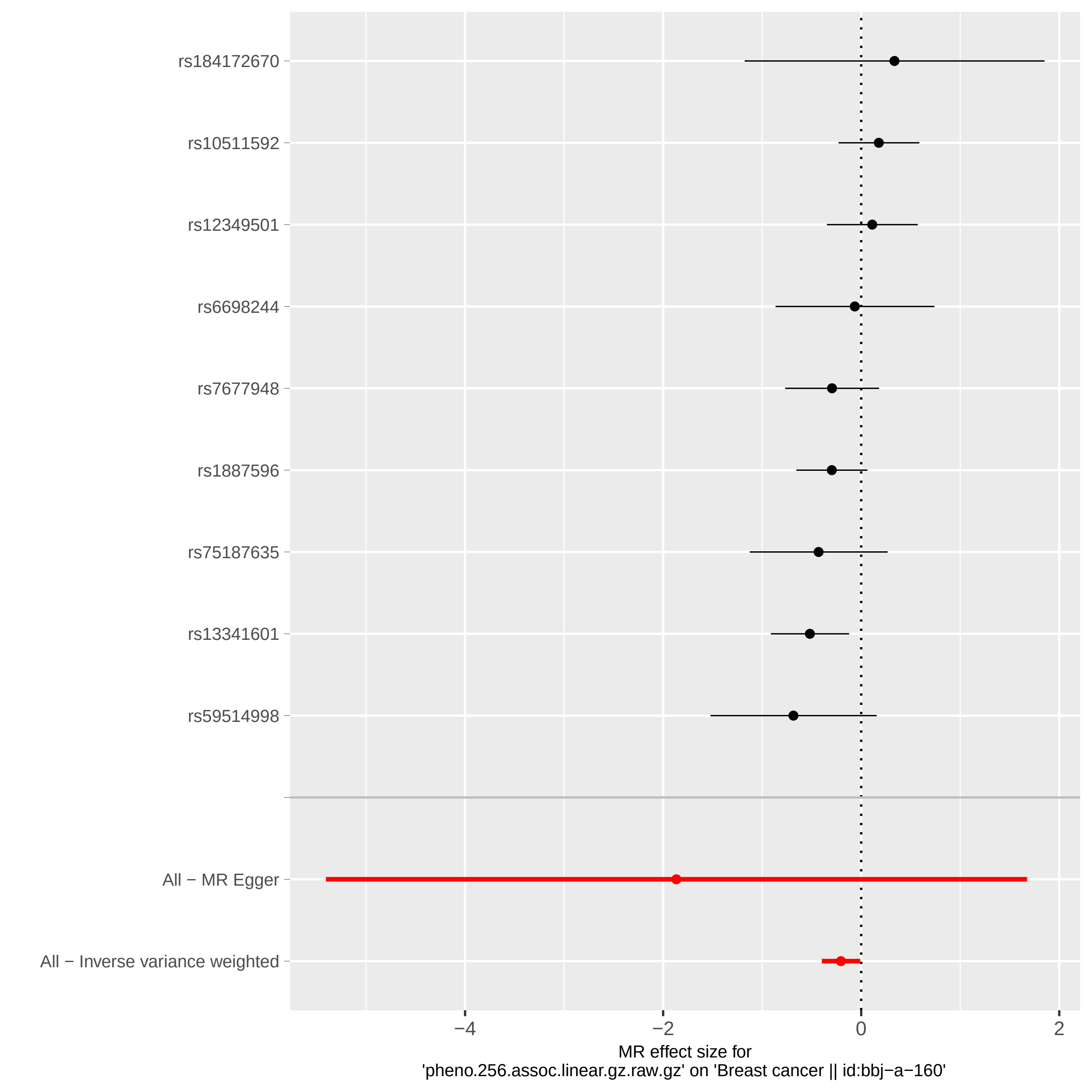
rs184172670
rs10511592
rs12349501
rs6698244
rs7677948
rs1887596
rs75187635
rs13341601
rs59514998
All − MR Egger
All − Inverse variance weighted
−4
−2
0
2
MR effect size for
'pheno.256.assoc.linear.gz.raw.gz' on 'Breast cancer || id:bbj−a−160'
